# CO_2_-responsive Pickering emulsions stabilized by soft protein particles for interfacial biocatalysis†

**DOI:** 10.1039/d1sc06146a

**Published:** 2022-01-18

**Authors:** Yongkang Xi, Bo Liu, Shuxin Wang, Shuheng Wei, Shouwei Yin, To Ngai, Xiaoquan Yang

**Affiliations:** Research and Development Centre of Food Proteins, School of Food Science and Engineering, Guangdong Province Key Laboratory for Green Processing of Natural Products Safety, South China University of Technology Guangzhou 510640 P. R. China feysw@scut.edu.cn; Department of Chemistry, The Chinese University of Hong Kong Shatin N. T. Hong Kong tongai@cuhk.edu.hk; Sino-Singapore International Joint Research Institute Guangzhou 510640 P. R. China; Research Institute for Food Nutrition and Human Health Guangzhou P. R. China

## Abstract

Pickering emulsions are emulsions stabilized by colloidal particles and serve as an excellent platform for biphasic enzymatic catalysis. However, developing simple and green strategies to avoid enzyme denaturation, facilitate product separation, and achieve the recovery of enzyme and colloidal particle stabilizers is still a challenge. This study aimed to report an efficient and sustainable biocatalysis system *via* a robust CO_2_/N_2_-responsive Pickering oil-in-water (o/w) emulsion stabilized solely by pure sodium caseinate (NaCas), which was made naturally in a scalable manner. The NaCas-stabilized emulsion displayed a much higher reaction efficiency compared with conventional CO_2_/N_2_-responsive Pickering emulsions stabilized by solid particles with functional groups from polymers or surfactants introduced to tailor responsiveness, reflected by the fact that most enzymes were transferred and enriched at the oil–water interface. More importantly, the demulsification, product separation, and recycling of the NaCas emulsifier as well as the enzyme could be facilely achieved by alternatively bubbling CO_2_/N_2_ more than 30 times. Moreover, the recycled enzyme still maintained its catalytic activity, with a conversion yield of more than 90% after each cycle, which was not found in any of the previously reported CO_2_-responsive systems. This responsive system worked well for many different types of oils and was the first to report on a protein-based CO_2_/N_2_-responsive emulsion, holding great promise for the development of more sustainable, green chemical conversion processes for the food, pharmaceutical, and biomedical industries.

## Abstract 2

An unprecedented strategy was proposed for recycled interfacial biocatalysis in a CO_2_-responsive emulsion stabilized by soft protein particles. The recycled enzyme maintained its catalytic activity, with a conversion yield over 90% after 30 cycles.

## Graphical abstract

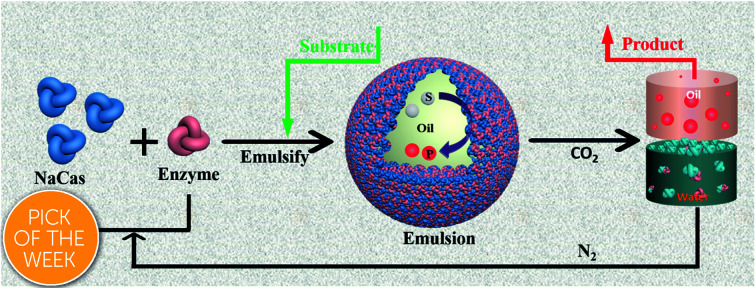

## Introduction

Enzymes are potent biocatalysts for a wide range of organic reactions, providing necessary chemicals and pharmaceuticals with high regio-, chemo-, and stereo-selectivity under sustainable and mild conditions.^1,2^ They routinely prefer an aqueous medium and show high activity therein, while most substrates in organic reactions are insoluble in water.^3,4^ Therefore, enzymatic reactions are typically performed in aqueous-organic biphasic systems, while they often show low catalysis efficiencies due to a limited interfacial area.^3^ A facile and effective strategy to address the issue is to generate emulsions that enlarge the interfacial area between an organic medium and an aqueous phase, thereby improving mass transfer properties and facilitating easy recovery of the emulsifier and enzymes.^1^ Indeed, it is a clear advantage if both the formation and demulsification of an emulsion can be controllably accessible. In this sense, environment-responsive emulsifiers, including surfactants, polymers, and solid particles, have recently attracted considerable interest because they not only have the ability to stabilize emulsions but also serve as a tool to engineer the emulsion by responding to environmental changes, such as pH,^5–7^ temperature,^8^ light,^3^ chemical agents,^9–11^ or even a combination of them. The use of stimuli-responsive emulsifiers to carefully control the emulsion structure and reversibility could effectively decrease waste generation, surfactant consumption, and process remediation costs, thus promoting the greenness of chemical conversion processes.^12^

Although an environment-responsive emulsion for biphasic biocatalysis can offer considerable advantages from a green chemistry perspective, the practical viability of switchable emulsifiers is challenging in biocatalysis because triggers should protect enzymes from damage, effectively drive system recycling, involve a simple procedure, and consume low energy. In retrospect, the most-studied approach has been pH- and thermo-responsive emulsions.^13–16^ However, the accumulation of salts usually occurs in pH-responsive systems, which may be deleterious to ionic strength-susceptible reactions. Thermoresponsive systems are nonaccumulative but energy intensive. In addition, these triggers may have irreversible or harmful influence on the activity of many enzymes. Most importantly, the formation of smart emulsifiers routinely requires chemical modification, which is cumbersome and wasteful, in particular when more advanced emulsifiers are required.

Recently, CO_2_/N_2_ triggers were reported to have promising features because both CO_2_ and N_2_ are typically biocompatible, widely available, low cost, environmentally friendly, and simple to implement.^17–20^ Furthermore, CO_2_ and N_2_ exhibit less harm to the activity of an enzyme because both of them are noncorrosive and nontoxic, and can also be facilely and easily got rid of from the system on demand.^21^ In this context, interest in Pickering interface catalysis with CO_2_/N_2_ triggers started a prairie fire.^22–26^ Little is known, however, about the performance of enzymes in CO_2_/N_2_-switchable emulsions. A noteworthy reason is that, very often, a CO_2_/N_2_-responsive architecture is designed by surface grafting with moieties that inherently have responsive features, for example, *N*′-dodecyl-*N*,*N*-dimethylacetamidine, 2-(dimethylamino)ethyl methacrylate and *N*,*N*-dimethyldodecylamine.^21,27,28^ In 2019, Yang and co-workers reported for the first time on CO_2_/N_2_-responsive emulsion biocatalysis, in which emulsions were co-stabilized by bare SiO_2_ nanoparticles and a CO_2_-responsive surfactant (*N*,*N*-dimethyldodecylamine).^21^ Recently, Wu and co-workers reported using a diblock copolymer to obtain pH-, CO_2_-, and thermo-responsive emulsions for sequential multienzyme cascades.^29^ Although these responsive emulsions were very versatile, the sensitive amphiphilic components, such as small surfactants or block polymers that are adsorbed/grafted on solid emulsifiers, were shown to weaken enzymatic activity, concomitantly challenging the streamlining workup and purification.^1,4,30–33^ Even worse, leaching or detaching of the grafted or adsorbed sensitive components on the solid emulsifiers led to a change in surface wettability or stimulus-responsive ability or loss of emulsifying performance. This explained why the reported CO_2_-switching emulsions were usually recycled several times and the enzymatic reaction decreased greatly in each cycle. Therefore, exploring efficient strategies for constructing sustainable and green CO_2_/N_2_-responsive emulsion biocatalytic platforms is ever-increasingly important to improve the efficiency and recyclability of an enzyme catalyst. In light of this, proteins have many advantages, such as good compatibility with enzymes, nontoxicity, high stability, and easy handling, which are not accessible for conventional inorganic and/or naturally organic colloidal particles. More importantly, they have strong adhesion to the interface that is intrinsic without adding/grafting any component. This behavior benefits not only the ease of preparation but also the long-term use of the material. So far, none of the studies reported on the construction of CO_2_/N_2_-responsive emulsions using proteins as effective emulsifiers.

**Scheme 1:**
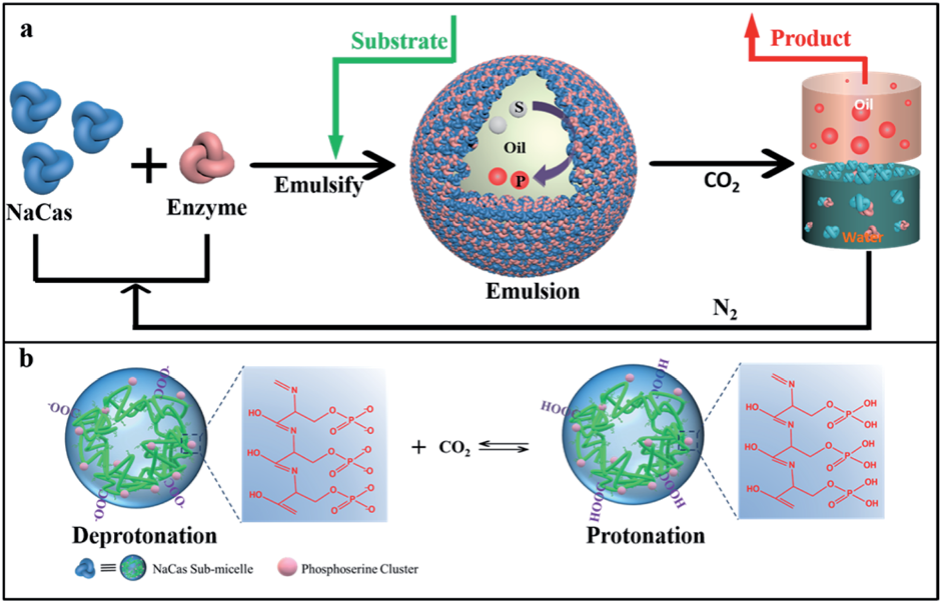
(a) Schematic illustration of recycled emulsion biocatalysis using a CO_2_/N_2_-responsive NaCas-stabilized emulsion platform for a recoverable catalyst in streamlining biocatalysis processes. (b) Schematic illustration of the CO_2_ switchability of NaCas. As CO_2_ was bubbled, the protonation of phosphine serine clusters and carboxyl groups reduced the electrostatic repulsion force, leading to the aggregation and flocculation of NaCas into disordered aggregates, resulting in the demulsification of the emulsion. In contrast, the initial morphology of NaCas was reversibly restored by adding N_2_ to remove CO_2_.

The aforementioned limitations prompted us to seek elegant systems, in which CO_2_/N_2_-responsive emulsions and biocatalysts can be integrated to facilitate the recovery and reuse of biocatalysts. In this study, an unprecedented method has been reported to construct CO_2_/N_2_-responsive emulsions stabilized solely by pure sodium caseinate (NaCas) to proceed with robustly recycled biocatalysis. Avoiding adding/grafting sensitive inorganic or organic moieties onto NaCas protein allowed carrying out efficient and safe enzymatic reactions that were highly suitable for the pharmaceutical, food, and cosmetic industries. As shown in Scheme 1, a facile but potent strategy was proposed for recycled biphasic biocatalysis in a CO_2_-responsive emulsion system. An enzymatic reaction was carried out on the as-prepared switchable emulsion platform by dispersing the *Candida antarctica* lipase B (CalB) lipase in water before emulsification. The emulsions were broken by introducing CO_2_ at room temperature after the reaction was completed. As a result, the products rich in the oil phase could be easily isolated and collected, and NaCas and the CalB enzyme in the water phase could be recycled. Interestingly, this responsive system worked well for many different types of oils, and all emulsions could be turned on or off by alternatively bubbling CO_2_ and N_2_ within a few minutes. Last but not least, the uniqueness of the switchable NaCas-stabilized emulsion was investigated through comparing the performance of emulsions stabilized by five representative proteins to clarify how NaCas worked at the oil–water interface in controlling the emulsion formation and demulsification. In addition, using this system in biocatalysis, enzymes were adsorbed onto the surface of emulsion droplets, facilitating the improvement of the performance of the enzyme reactions without incompatibility issues.

## Results and discussion

Casein represents a family of intrinsically disordered proteins, including α-casein (α_s1_- and α_s2_-caseins), β-casein, and k-casein. Of these, α_s1_-casein is a triblock biopolymer, while β-casein and k-casein were monomeric biopolymers with hydrophilic and hydrophobic extremities. NaCas, a derivative of casein, is a robust amphipathic protein with a highly disordered structure. NaCas could aggregate into spherical micelles (sub-micelles) under neutral and/or basic conditions.^34–36^ In this study, CO_2_/N_2_-switchable emulsions were constructed using pure NaCas as the sole emulsifier and the emulsion-inversion ability was examined.

**Fig. 1:**
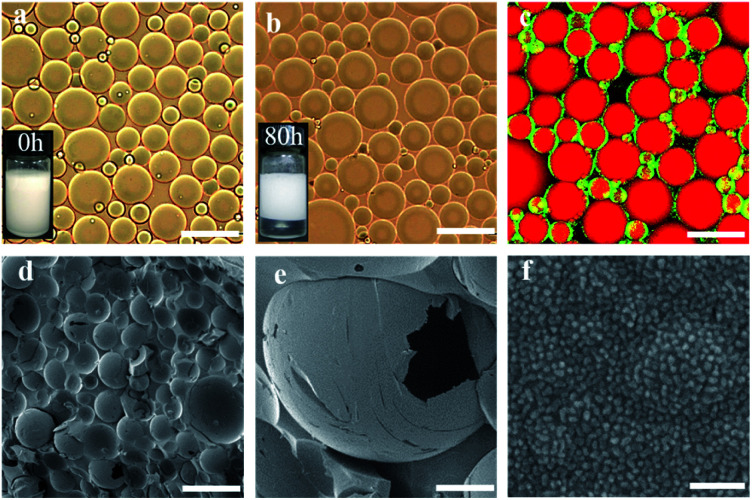
CO_2_/N_2_-switchable emulsions stabilized with 0.1 wt% NaCas. Optical micrographs and visual appearance of the emulsions using *n*-heptane as the oil phase after storage for 0 h (a) and 80 h (b). CLSM image (c) and cryo-SEM image (d–f) of the emulsions with *n*-heptane as the oil phase. Scale bar: (a–d) 100 μm, (e) 30 μm, and (f) 500 nm.

CO_2_/N_2_-responsive emulsions were solely stabilized by NaCas. A typical o/w emulsion was observed after emulsifying the mixture consisting of an equal volume of *n*-heptane and 0.1 wt% of NaCas solution by shaking or homogenization (15 000 rpm for 1 min). The emulsions were characterized as spherical droplets with a size of 20–50 μm (Fig. 1a and c–e), by optical microscopy, cryo-scanning electron microscopy (cryo-SEM), and confocal laser scanning microscopy (CLSM). In Fig. 1f, the cryo-SEM images reveal that the surface of emulsion droplets was composed of closely packed colloidal particles of NaCas (diameter 20–40 nm, which was consistent with the observed transmission electron microscope (TEM) image of NaCas; Fig. 4e). In addition, the NaCas emulsions were stable for at least 80 h (Fig. 1b).

**Fig. 2:**
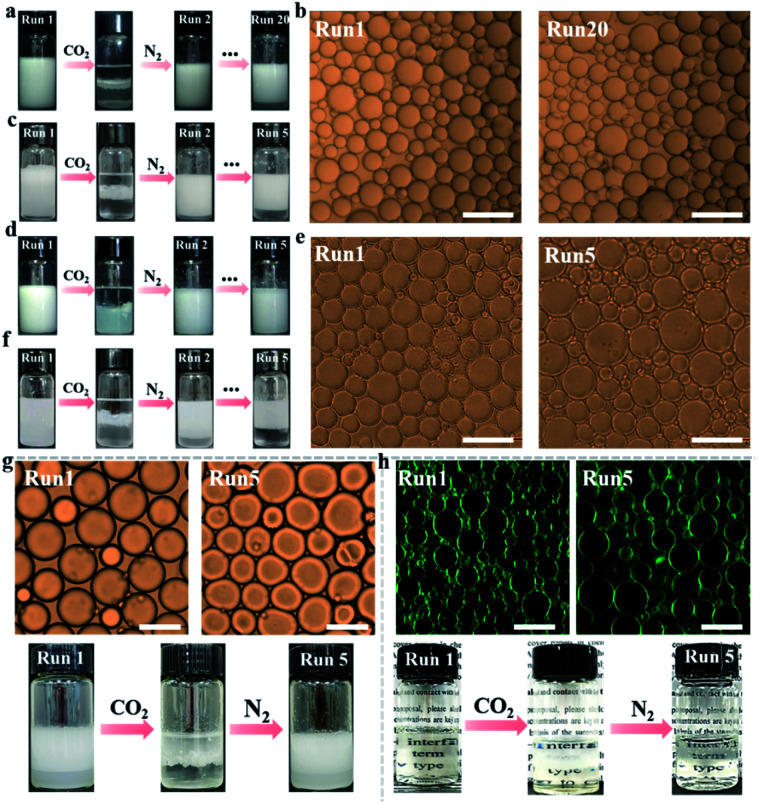
Optical micrographs and appearance of successive conversion cycles of the CO_2_/N_2_-responsive NaCas-stabilized emulsion (0.1 wt%) using *n*-heptane (a and b), ethyl acetate (d and e) and squalene (g) as the oil phase. (c) Appearance of NaCas-stabilized emulsion conversion cycles using a 50 mM salt solution as the aqueous phase and *n*-heptane as the oil phase. (f) Appearance of NaCas-stabilized emulsion conversion cycles using a 5 M salt solution as the aqueous phase and ethyl acetate as the oil phase. (h) CLSM and appearance of successive CO_2_/N_2_-responsive NaCas-stabilized transparent emulsion (0.1 wt%) conversion cycles using *n*-octane. Scale bar: 100 μm.

Interestingly, bubbling CO_2_ resulted in complete macroscopic phase separation of the emulsions stabilized by NaCas within 1 min. As expected, this system restored the emulsion by shearing the oil–water mixture after CO_2_ was removed from the mixture by bubbling N_2_. Impressively, this CO_2_ switchable emulsion could be reversibly switched on and off over 20 cycles (Fig. 2a), which could not be achieved for emulsions co-stabilized using silica nanoparticles *in situ* decorated with a CO_2_/N_2_-switchable surfactant. The emulsifying and responsive performance of NaCas remained unaffected throughout the cycles because the type and particle sizes of the regenerated emulsions were similar to the equivalents of the raw ones (Fig. 2b). Salts usually passivate the cyclic system by promoting the flocculation of emulsifiers. Therefore, the effects of salt concentrations on the as-prepared CO_2_/N_2_-triggered responsive system were investigated. Interestingly, the CO_2_/N_2_-responsive cycles worked well in 50 mM NaCl for at least five runs (Fig. 2c), implying that a robust and extraordinary CO_2_/N_2_-triggered switchable emulsion platform was established with NaCas protein as the particulate emulsifier.

### Universality and potential applications

Besides the *n*-heptane/water and ethyl acetate/water binary systems, NaCas was also found to be a good switchable emulsifier for stabilizing reversibly a series of oils with an interface tension between 1.8 and 51.1 mN m^−1^, reflecting that this strategy was generic, robust, and versatile (Fig. 2d, S1† and Table 1). In these systems, all emulsions could be switched on or off through alternately bubbling CO_2_/N_2_. Demulsification occurred perfectly within 1 min during bubbling CO_2_. Most importantly, the CO_2_/N_2_-switchable process functioned well after five cycles with a broad range of oils as the oil phase. Clearly, the type and particle sizes of the emulsions did not change after the cycles (Fig. 2e). The as-prepared emulsion also functioned well in the presence of a high salt concentration. For example, the process functioned for at least five runs in the presence of 5 M salt with ethyl acetate as the oil phase (Fig. 2f).

**Table 1:** Interfacial characteristics and polarity of various oils used for constructing CO_2_/N_2_-responsive emulsions^7,37^

Oil phase	*γ*_ow_ (mN m^−1^)	Polarity	Transferable
*n*-Hexane	51.1	0.06	Yes
*n*-Heptane	49.4	0.2	Yes
Dichloromethane	44.3	3.4	Yes
Petroleum ether	42	0.01	Yes
Toluene	35.7	2.4	Yes
Benzene	35	3	Yes
Ethyl acetate	19.8	4.3	Yes
*n*-Butanol	1.8	3.7	Yes

Next, we further extended the system to produce submicroemulsion and transparent emulsions, which could also be switched on and off on demand. In some cases, responsive submicroemulsion facilitates application in topical formulae for transdermal drug delivery because of its ability to solubilize substantial amounts of hydrophobic drugs exerting therapeutic functions on command. As shown in Fig. S2,† NaCas-stabilized isopropyl myristate submicroemulsion rapidly destabilized and released indomethacin after CO_2_ addition. In addition, we showed that a responsive transparent emulsion could be created with NaCas as the sole emulsifier, in which the transparency was regulated by the sucrose content. Interestingly, this system could be reversibly turned on and off over five cycles after alternately bubbling CO_2_/N_2_ (Fig. 2h and S3†).

In light of the excellent suitability of the aforementioned system and its safety because of containing proteins only, this reversible CO_2_/N_2_ convertible emulsion system is expected to be widely used in crude oil extraction and cosmetics (targeted release of active ingredients on command). For example, the controlled release of squalene from cosmetics and the efficient recovery of kerosene were achieved (Fig. 2g and S4†).

### Specificity of CO_2_/N_2_-responsive emulsions was stabilized using NaCas

**Fig. 3:**
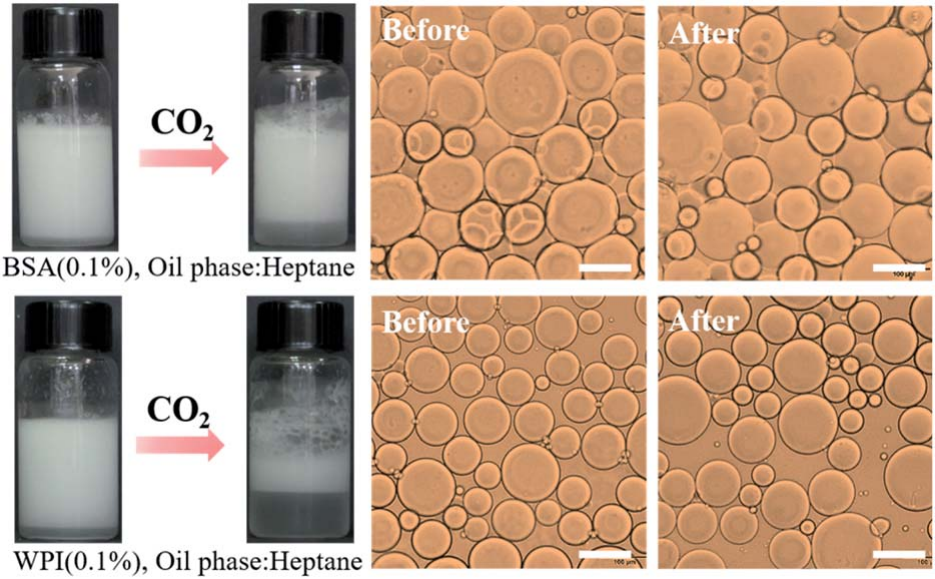
CO_2_/N_2_-responsive behavior of emulsions stabilized using different types of proteins. Scale bar: 100 μm.

How NaCas-stabilized emulsions could be cycled using CO_2_/N_2_ as the trigger? To answer this question, a wide range of emulsions were constructed with five selected proteins as the emulsifier, including four animal proteins whey protein isolate (WPI), bovine serum albumin (BSA), lactoferrin, and gelatin, and one plant protein soy protein isolate (SPI). The CO_2_/N_2_-stimuli switchability of the above-mentioned emulsions was investigated. In brief, the protein solution was blended with *n*-heptane as the oil phase at a volume ratio of 1 : 1, and the resultant mixtures were emulsified by shearing for 1 min at a rate of 15 000 rpm. The CO_2_/N_2_-switchable performance of the emulsions was then checked. Unfortunately, the emulsions stabilized by WPI, BSA, lactoferrin, and SPI did not break when the as-prepared emulsions were bubbled with CO_2_, and unstable emulsions were produced with gelatin using the aforesaid procedure (Fig. 3 and S5†). We thereby surmised that the unique CO_2_/N_2_-responsive behavior of NaCas-stabilized emulsions should be mainly attributed to the specific protein structure of NaCas.

### Working principle of CO_2_/N_2_-responsive emulsions stabilized with NaCas

**Fig. 4:**
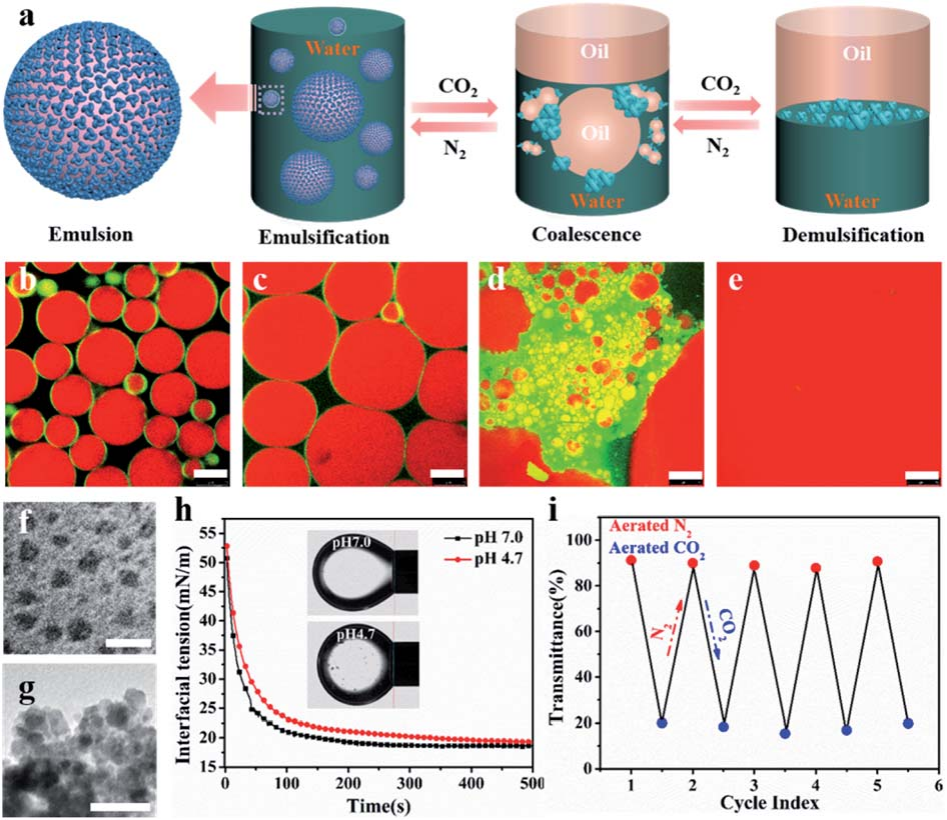
CO_2_/N_2_-responsive emulsions stabilized by NaCas. (a) A schematic diagram on the working principle of the CO_2_/N_2_-responsive emulsions. CLSM image of the emulsions using *n*-heptane as the oil phase at pH 6.8 (b), 6.0 (c), 5.5 (d), and 4.7 (e). Morphological evolution of NaCas triggered by bubbling CO_2_/N_2_: TEM image at pH 6.8 (f) and 4.7 (g). (h) The evolution of interfacial tension with time at various pH values using dodecane as the oil phase. (i) Reversible transmittance changes in NaCas solution upon cycling between introducing CO_2_ and N_2_. Scale bar: 25 μm (b and c), 100 μm (d and e), and 50 nm (f and g).

CLSM was used to characterize the NaCas behavior at the oil–water interface to understand the specificity of the switchable emulsion stabilized by NaCas. Fig. 4b shows that small droplets with a complete interface layer were clearly visualized, reflecting that these droplets had higher interface coverage under the initial conditions. As CO_2_ was bubbled, the zeta potential decreased gradually due to the protonation of the phosphate serine cluster and carboxyl, which enhanced the hydrophobic attraction between emulsifiers, resulting in the aggregation and flocculation of NaCas into disordered aggregates. Emulsion coalescence and even demulsification were thus observed in Fig. 4c–e. Interfacial adsorption was conducted for NaCas at different pH values to gain more insight (Fig. 4h); NaCas had a similar interfacial tension (19.31 *vs.* 18.92 mN m^−1^) and adsorption rate (4.92 *vs.* 5.77 mN m^−1^ S^−0.5^) at pH 4.7 and 7.0, implying that the evolution in the interfacial activity of the emulsifier did not account for CO_2_-trigged demulsification of the NaCas emulsion. Moreover, reversible CO_2_/N_2_-triggered association–disassociation of NaCas further explained the working principle of the emulsion. Typically, NaCas aggregated into spherical micelles with diameters of 20–40 nm at neutral pH (Fig. 4f). They evolved into a cluster-like structure by bubbling CO_2_, accompanying the pH shift to 4.7, and finally precipitated upon further bubbling of CO_2_. Impressively, the association was perfectly reversible when CO_2_ was washed off by introducing N_2_, and the pH value of NaCas solution returned to 7.0 (Fig. 4i). Fig. S6† confirms that the nanoarchitecture of NaCas protein remained unaffected even after six cycles. In particular, the colloidal architecture was formed at pH 4.7 by the flocculation of sub-micelles of 20–40 nm, but did not coalescence or merge (Fig. 4g). A schematic diagram is proposed to make clear the working principle of the CO_2_/N_2_-responsive emulsion system (Fig. 4a).

**Fig. 5:**
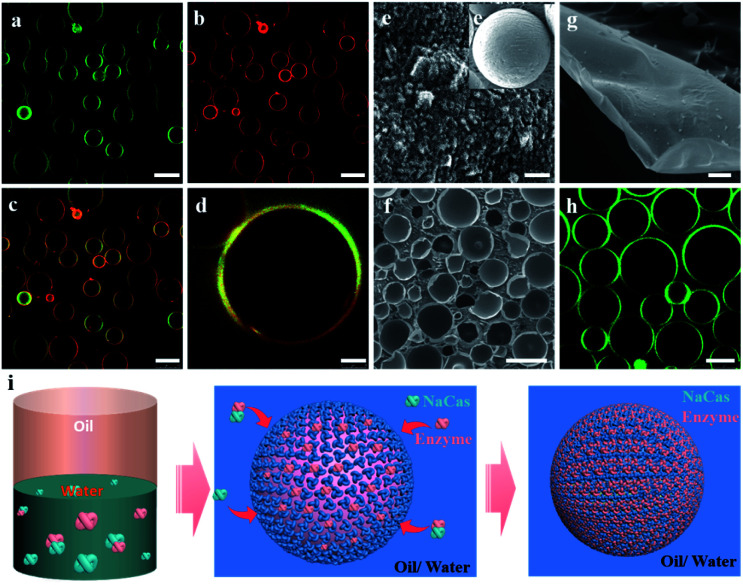
CLSM image of the emulsion stabilized by a mixture of 0.1 wt% NaCas labeled with fluorescein isothiocyanate (FITC) (a) and 0.5 mg mL^−1^ CalB labeled with Cy5 (b). (c and d) Overlay image of NaCas and CalB fluorescence signals. Cryo-SEM images of NaCas–CalB-stabilized emulsions at neutral pH (e and e_1_) using *n*-dodecane as the oil phase. Cryo-SEM images of NaCas–CalB-stabilized emulsions at neutral pH (f and g) using *n*-heptane as the oil phase. (h) CLSM image of the emulsion stabilized by a mixture of 0.1 wt% NaCas and 0.5 mg mL^−1^ cytochrome c labeled with FITC. (i) Schematic illustration of enzyme transport in the NaCas-stabilized CO_2_/N_2_-responsive emulsion system. Scale bar: 100 μm (a–c), 25 μm (d and h), 200 nm (e and e_1_), and 1 μm (g).

Thus, a robust CO_2_-responsive emulsion system stabilized solely by NaCas was established, which could work with all sorts of oils, providing facile and quick demulsification, as well as robustly switchable cyclability, even in complicated and sensitive circumstances, for example, a salt solution. These responsive emulsion systems helped construct an efficiently recyclable biocatalysis platform. However, the location of catalysts is critical to its performance in emulsion catalysis. Therefore, the enzyme and NaCas were labeled with Cy5 (excitation wavelength 635 nm) and fluorescein isothiocyanate (FITC, excitation wavelength 488 nm), respectively, to clarify the partition of the enzyme within the responsive emulsion. As shown in Fig. 5a and b, the green (NaCas) or red fluorescence (CalB) with a strong signal was concentrated around spherical droplets, indicating that most of the enzyme molecules were of targeted enrichment at the oil–water interface during the emulsification by the delicate interaction between NaCas and the enzyme (Fig. S7a and b†). Interestingly, these two signals basically overlapped, indicating that the enzyme was embedded in the film formed by NaCas at the oil–water interface (Fig. 5c and d). The cryo-SEM images of the emulsion also showed essentially the same surface morphology comprising closely packed particles with particle sizes of 20–40 nm (Fig. 5e and e1). In addition, the colloids comprising these interface shell structures (Fig. 5f and g, average thickness of 50 nm) were highly ordered. Subsequently, the unique film at the oil–water interface might be quite advantageous for the enzyme molecules to come into contact with the substrate, helping to enhance the subsequent catalytic reaction. The interfacial activity of lysozyme and cytochrome c and their distribution in the NaCas-stabilized emulsion were also determined using an interfacial tension meter and CLSM to further validate our strategy. As shown in Fig. S8,† the interfacial activity of the enzyme was much lower than that of NaCas, resulting in an inability to form a stable emulsion because of their low interfacial activity (Fig. S9†). Quite impressively, the enzyme labeled with Cy5 could be detected at the oil–water interface of the emulsion and presented a green fluorescent ring around the droplets, confirming that the enzyme tightly wrapped the surface of the emulsion droplets (Fig. 5h and S7c†).

On the basis of these results, it was concluded that these enzymes were successfully transported to the oil–water interface in the NaCas-stabilized emulsion. This unique membrane at the oil–water interface might be very beneficial for the enzyme molecules to come into contact with the substrate. Compared with the free enzyme in the two-phase system, the enzyme adsorbed at the oil–water interface maximized the range of the catalyst–liquid interface and promoted the mass transfer between the two phases, which helped enhance the subsequent catalytic reaction. Moreover, this system might offer several advantages over solid particle stabilized emulsions in catalysis. For example, it not only reduced the high energy consumption in product and catalyst recovery but also avoided the effect of tightly packed nanoparticles on the mass diffusion of the substrate [2b].

**Fig. 6:**
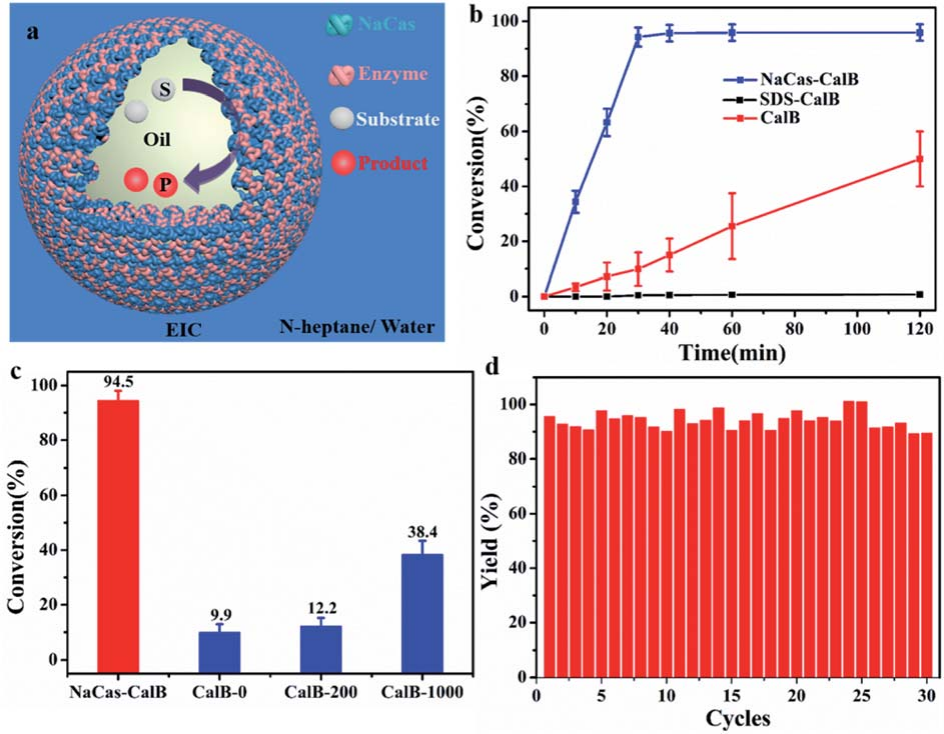
(a) Biphasic biocatalytic pathway in the emulsion stabilized by NaCas and an enzyme. (b) Plot of the conversion of the esterification between hexanoic acid and 1-hexanol catalyzed by CalB *versus* reaction time. Two phases: CalB in a biphasic water/toluene system; sodium dodecyl sulfate (SDS)–enzyme: an emulsion stabilized by a mixture of 0.05 wt% SDS and CalB (0.5 mg mL^−1^); NaCas–CalB: an emulsion stabilized by a mixture of 0.1 wt% NaCas and CalB (0.5 mg mL^−1^). (c) Esterification conversion of CalB (0.5 mg mL^−1^) as a function of stirring speed in a two-phase reaction. (d) Recycling results for the esterification yield by interfacial CalB in the *n*-heptane/water system. The reaction time was 30 min.

The interfacial targeted enrichment of biocatalysts enabled subsequent construction for interfacial biocatalysis. The esterification reaction between hexanoic acid and 1-hexanol was used as the model reaction to assess the catalytic performance of the enzyme on a CO_2_-responsive emulsion-based biphasic biocatalysis platform (Fig. 6a). Three strategies were used to perform the biocatalysis reaction, and CalB was incorporated differently. The first approach was a two-phase system without emulsification, which is a typical system in the industry. Conventional emulsions stabilized by the surfactant (SDS) were developed as the second approach. The third approach was a responsive emulsion stabilized by CO_2_-switchable NaCas. These three systems possessed the same reaction conditions, such as the amount of lipase, the substrate concentration, and the ratio of oil-to-water. As shown in Fig. 6b, after 30 min, the NaCas–enzyme system achieved 94.5% conversion of esters (Fig. S10 and S11†), but a few products were detected in the two-phase reaction system (9.9%). The higher enzymatic activity of the emulsion reaction over the two-phase reaction might be attributed to the significant increase in the interfacial area with the emulsion droplets as microreactors (increased by at least 492 times), thus contributing to faster biotransformation. Furthermore, compared with the conversion of 0.47% achieved in the SDS–enzyme emulsion system, the excellent performance achieved by the NaCas–enzyme emulsion system further demonstrated the significance of natural emulsifiers in enzyme catalysis. Surfactants such as SDS can easily damage the enzymes, which can induce unfolding of the enzyme. The conversion of the esterification was compared between the NaCas–enzyme emulsion system and the two-phase system with traditional stirring to further justify the odds of this system. Surprisingly, a nearly 2.4-fold increase in conversion was recorded in the NaCas–enzyme emulsion system than in the two-phase system (Fig. 6c, conversion 38.4%, up to 1000 rpm).

Besides the catalytic activity of the enzyme, its recyclability is another critical parameter affecting the practical application of the enzyme because it is susceptible and relatively expensive. Therefore, the recyclability of the enzyme was tested on the CO_2_-responsive emulsion biocatalysis platform. After the reaction, the emulsions were broken by bubbling CO_2_ for 1 min at ambient temperature (Fig. 6d). After demulsification, the product existed in the oil phase while both the enzyme and NaCas were in the aqueous phase. Then, the upper oil phase was separated by simple decantation to collect the product. The aqueous phase was used for directing the next reaction cycle. The recycled aqueous phase was mixed with fresh *n*-heptane with the substrate. The emulsion was restored by bubbling N_2_ through the resulting mixture for 10 min at room temperature, followed by shearing as described previously, and the enzymatic reaction was performed as earlier. The enzyme reaction in CO_2_-responsive emulsions was successfully re-performed over 30 consecutive runs, and the conversion was similar and more than 90% in each case. Therefore, we succeeded in constructing robustly recycled emulsions with high recyclability and stability (Fig. 6d, S12 and S13†). Compared with other separation methods for isolating and recycling the emulsifiers and enzymes such as centrifugation or ultrafiltration, our N_2_/CO_2_ switchable strategy enabled a scalable operation, which did not need expensive or special instrument. The demulsification and recycling of the emulsifier and enzyme could be realized by introducing CO_2_ at room temperature. These results confirmed the superiority of the proposed strategy in terms of enzyme catalysis using NaCas other than surfactant analogs for emulsion biocatalysis.

## Conclusion

In summary, this study demonstrated the first successful use of pure NaCas as the sole emulsifier for constructing a robust CO_2_/N_2_-responsive emulsion to proceed with recycled biocatalysis, which allowed overcoming the limitations of the existing methods and efficiently bridging the gaps between efficient catalysis and catalyst recovery in an unprecedented manner. The recovery technique itself was fairly easy to process, that is, it only needed bubbling with CO_2_, thus obviating the enzyme deactivation and sophistication process. Since the NaCas response emulsion successfully transferred the enzyme to the oil–water interface by physical adsorption, it can be expected that it allows reversible immobilization because the immobilized enzymes can be released from the interface into the aqueous phase when the NaCas-stabilized emulsion droplets are demulsified. This is definitely beneficial to the recovery of various enzymes in the food and pharmaceutical industries *via* using an economic technology. Moreover, the novel idea or protocol for the use of NaCas as a smart emulsifier not only is significant for creating robust switchable emulsions extensively applied as biocatalysis platforms but also makes it possible to develop controlled delivery of drugs and cosmetics, which is a big step forward for the green chemistry of the emulsion and colloid area.

## Data availability

All experimental supporting data and procedures are available in the ESI.†

## Author contributions

S. W. Y. and T. N. conceived and managed the research. Y. K. X., B. L., S. X. W. and S. H. W. performed the reversible CO_2_-responsiveness measurement of NaCas, responsive emulsion preparation and characterization, biocatalysis, figure preparation and data analysis. S. W. Y., T. N., and X. Q. Y. reviewed the results and provided the technical guidelines. Y. K. X., S. W. Y., and T. N. wrote the paper with input from B. L., S. X. W., and S. H. W. and all authors commented on the manuscript.

## Conflicts of interest

There are no conflicts to declare.

## Supplementary Material

SC-013-D1SC06146A-s001
