# Supplementary material for: CO2-responsive Pickering emulsions stabilized by soft protein particles for interfacial biocatalysis
Source: Chem Sci. 2022 Jan 18;13(10):2884–90. doi: 10.1039/d1sc06146a (PMC8905849; doi:10.1039/d1sc06146a)
Supplement: SC-013-D1SC06146A-s001 [file SC-013-D1SC06146A-s001.pdf]

Electronic Supplementary Material (ESI) for Chemical Science.
This journal is © The Royal Society of Chemistry 2022

Supporting information

CO_2-Responsive Pickering Emulsions Stabilized Solely by Green and Sustainable Protein Particles for Biphasic Biocatalysis

Yongkang Xi,[a] Bo Liu,[a] Shuxin Wang,[a] Shuheng Wei,[a] Shouwei Yin,*[a b, c] To Ngai*[b] and Xiaoquan Yang[a]

[a] Research and Development Centre of Food Proteins, School of Food Science and Engineering, and Guangdong Province Key Laboratory for Green Processing of Natural Products Safety, South China University of Technology, Guangzhou, 510640, P. R. China.

[b]Department of Chemistry, The Chinese University of Hong Kong, Shatin, N. T., Hong Kong.

[c]Overseas Expertise Introduction Center for Discipline Innovation of Food Nutrition and Human Health (111 Center), Guangzhou 510640, PR China

*Corresponding authors:

*Yin, S. W. E-mail: feysw@scut.edu.cn

*Ngai, T. E-mail: tongai@cuhk.edu.hk

Experimental Procedures

Materials.

NaCas was provided by Zhengzhou Taber Trading Co. Ltd. BSA was purchased from Beijing Dingguo Changsheng Biotechnology Co. Ltd (Beijing, China). WPI (NZMP) was purchased from Fonterra (New Zealand). SPI was extracted by previous report. Fluorescent dyes including Nile Red, Nile Blue A, cy5 and fluoresceine 5(6)-isothiocyanate mixed isomers (FITCs) were obtained from Sigma-Aldrich, Inc. (St. Louis, MO, USA). All other chemicals were of analytical grade.

CO_2/N_2-Responsive Performance of NaCas.

To investigate the CO_2/N_2-responsiveness of NaCas in aqueous solution, 5 mg of NaCas was dispersed in 10 mL of deionized water. The solution was repeatedly adjusted to pH 7.0 and 5.1 by the addition of CO_2 and N_2. The light transmittance and morphology of the NaCas solutions were monitored throughout the cycle. The pH of the NaCas solution was gradually adjusted to 4.7, and the morphology of NaCas was monitored using TEM or SEM.

In brief, TEM observations were performed using a JEM-1400 Plus (JEOL Ltd, Japan) at an acceleration voltage of 120 kV. The sample (0.005-0.05 wt%) was dispersed in deionized water, and the pH of the solution was adjusted to 6.8 or 4.7. A drop of the dispersion was spread onto 300 mesh copper grids coated with a carbon support film, and the specimens were then dried under vacuum for one day. In addition, a drop of phosphotungstic acid was added to stain NaCas for easy observation.

CO_2/N_2-Responsive Performance of Emulsions Stabilized by NaCas.

Typically, 10 mL of oil phase and 10 mL of a NaCas suspension (0.1 wt%) were mixed in 25 mL glass vessels at room temperature. Emulsions were formed using an Ultraturrax T10 homogenizer (IKA, Germany) at a stirring rate of 15 000 rpm for 1 min. After standing at room temperature for 30 min, a few CO_2 were added to adjust the pH to 4.6-5.0 to break up the emulsion. Finally, the cyclability of the NaCas-stabilized emulsions was assessed with a salt solution (NaCl 50mM and 5M) used as the solvent.

Transparent emulsion: 10 mL of oil phase and 10 mL of a NaCas suspension (contains sucrose) were mixed in 25 mL glass vessels at room temperature. Emulsions were formed using an Ultraturrax T10 homogenizer (IKA, Germany) at a stirring rate of 15 000 rpm for 1 min. After standing at room temperature for 30 min, a few CO_2 were added to adjust the pH to 4.6-5.0 to break up the emulsion.

Submicroemulsion: 5 mL of oil phase and 10 mL of a NaCas were mixed at room temperature. Then, high pressure microfluidics were used for four times emulsification under 50 Mpa to prepare submicroemulsion.

The morphology of the emulsion was characterized using an optical microscope, a laser confocal microscope and a cryo-electron microscope.

To verify the uniqueness of the pH-responsive NaCas-stabilized emulsions, we constructed a series of emulsions using 5 representative proteins, including four animal proteins (BSA, WPI and, gelatin and lactoferrin) and one plant proteins (SPI), and the CO_2/N_2-triggered cyclability of the emulsions were investigated.

Cryo-SEM measurements were performed on a Hitachi S-4800 instrument operating at 2.0 kV. Samples

(emulsions) were frozen with liquid nitrogen and sublimated for 30 min at −90 °C to expose the sample structure. The surface of the sample was removed with a blade to facilitate observation of the interfacial structure and immediately measured.

CLSM images were recorded on a Leica TCS SP5 confocal laser scanning microscope (Leica Microsystems Inc., Heidelberg, Germany) equipped with a 20-HC PL APO/0.70NA oil-immersion objective lens. The samples were stained with a mixture of Nile blue (0.1%) and Nile red (0.1%). The stained samples were placed on concave confocal microscope slides and examined using a 630x magnification lens. The two dyes were excited with an argon/krypton laser and a helium/neon (He-Ne) laser at 488 nm and 633 nm, respectively.

An optical contact angle meter (OCA-20) with oscillating drop accessory ODG-20 (Dataphysics Instruments GmbH, Germany) was used to measure the adsorption kinetics of NaCas solution at pH 7.0 and pH 4.7 (0.5 mg mL^{-1}). A drop of NaCas solution (10 μL) was delivered by an inverted tip of syringe into an optical glass cuvette containing purified oil, and allowed to stand for 500s to achieve adsorption at the oil-water interface. The drop image was photographed using the high-speed video camera, and the interfacial tension (γ) was calculated from the drop shape by Young–Laplace equation. All the measurements were performed in duplicates at a room temperature of 25 °C. For each sample two consecutive measurements were performed, and each sample was replicated at least 2 times.

Assessment of the Catalytic Performance of the Enzyme.

The catalytic performances of the lipases were evaluated in the esterification of 1-hexanol with hexanoic acid. Typically, the free lipase (0.5mg/mL) were dispersed into 10mL NaCas solution(0.1%), followed by addition of 10mL N-heptane(containing 50mM 1-hexanol and hexanoic acid). A stable emulsion was obtained by shaking 1min or shearing at 15,000 rpm for 1 min. For two-phase, except for the NaCas, the formulation, including the solvent composition, substrate concentration, and lipase content, were all the same as above. The esterification reactions were performed in the static state at 30 °C. Aliquots (25 μL) were extracted at different times and analyzed by gas chromatography (GC).

Results and Discussion

Universality and potential applications of this strategy

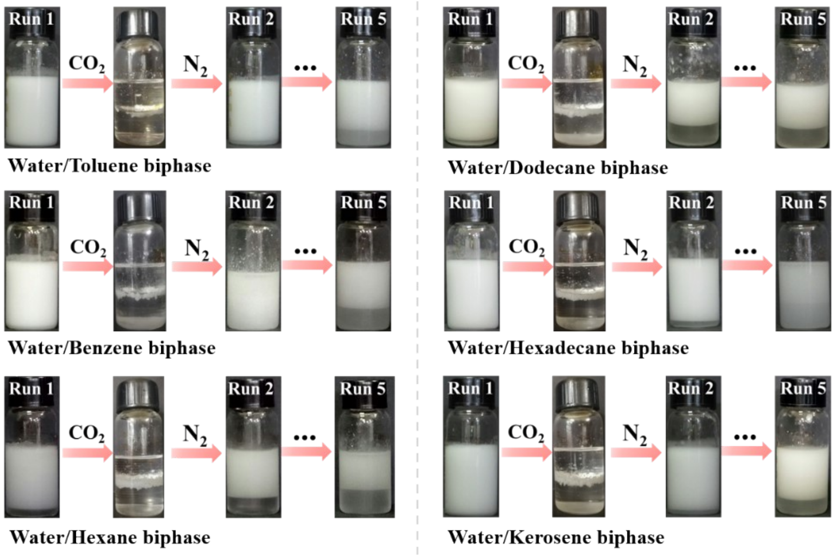

Figure S1. CO_2/N_2-responsive emulsions stabilized by NaCas. Appearance of successive CO_2/N_2-responsive NaCas-stabilized emulsion (0.1 wt%) inversion cycles using different oil (1st, 2nd and 5th cycle). After addition of an equal volume of oil phase into water containing 0.1 wt % of the sample (with respect to water), and subsequent homogenizing at a rate of 15 000 rpm for 1 min or shaking, different phenomena were observed for these samples. The as-prepared emulsion stabilized by NaCas was stable at neutral, and complete macroscopic phase separation occurred within 1 min upon introducing CO_2. This system, however, rapidly restored the emulsion (o/w) when N_2 were introduced and the pH of the water phase was adjusted to neutral. Remarkably, this emulsion could be reversibly switched on and off over 5 cycles.

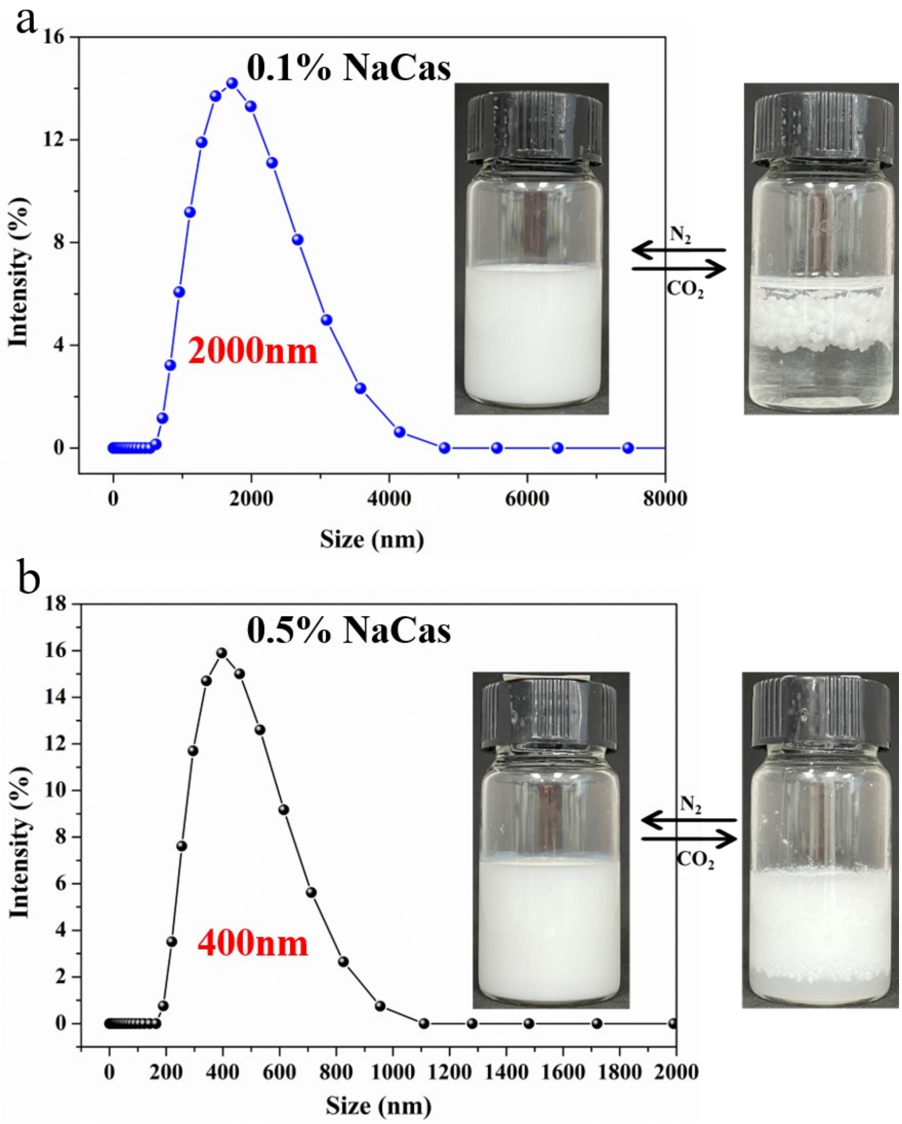

Figure S2. Size distribution and appearance of emulsion. (a) Size distribution and appearance of successive CO_2/N_2-responsive NaCas-stabilized emulsion (0.1 wt%, the emulsion size is small, but the emulsion has a good responsiveness on behalf of the size of the emulsion in the range of 1-4 μm.) conversion cycles using isopropyl myristate as the oil phase （oil to water=1:2). (b) Size distribution and appearance of successive CO_2/N_2-responsive NaCas-stabilized submicroemulsion (0.5 wt%) conversion cycles using isopropyl myristate as the oil phase (oil to water=1:2). These two emulsions were perpared by high-pressure microfluidics, which was homogenized for 4 times under 50 MPa.

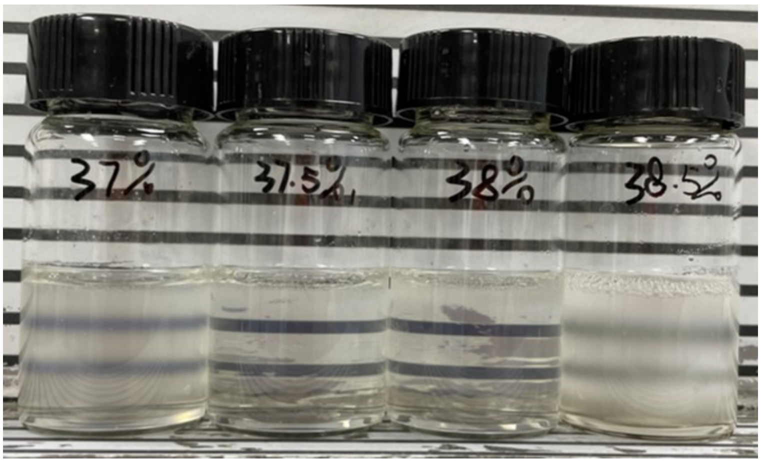

Figure S3. NaCas-stabilized transparent emulsion with different sucrose content. The refractive index of octane is about 1.3974 at 25 ℃. When sucrose concentration is 37.5%, the refractive index of sucrose aqueous solution (water phase) is the same as that of octane (oil phase), so the emulsion is transparent. This finding are of interest for providing a facile strategy to fabricate biocompatible and highly transparent responsive emulsion with great potential in the food, healthcare and light-induced catalysis fields.

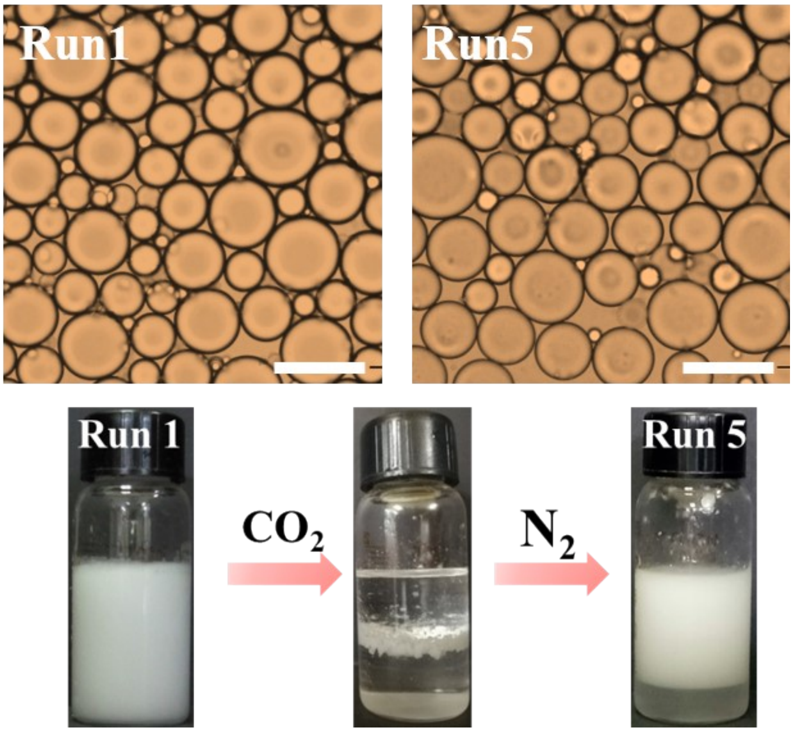

Figure S4. Optical micrographs and appearance of successive CO_2/N_2-responsive NaCas-stabilized emulsion (0.1 wt%) conversion cycles using kerosene as the oil phase. The as-prepared emulsions could be switched on or off through the bubbling of the CO_2/N_2. The type and droplet size of the regenerated emulsions remained almost the same during and after 5 cycles. Scale bar, 100 μm.

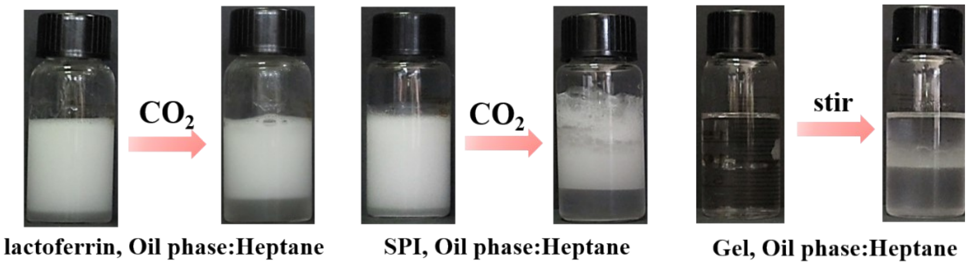

Figure S5. CO_2/N_2-responsive behaviour of emulsions stabilized by lactoferrin, SPI and Gelatin, respectively.

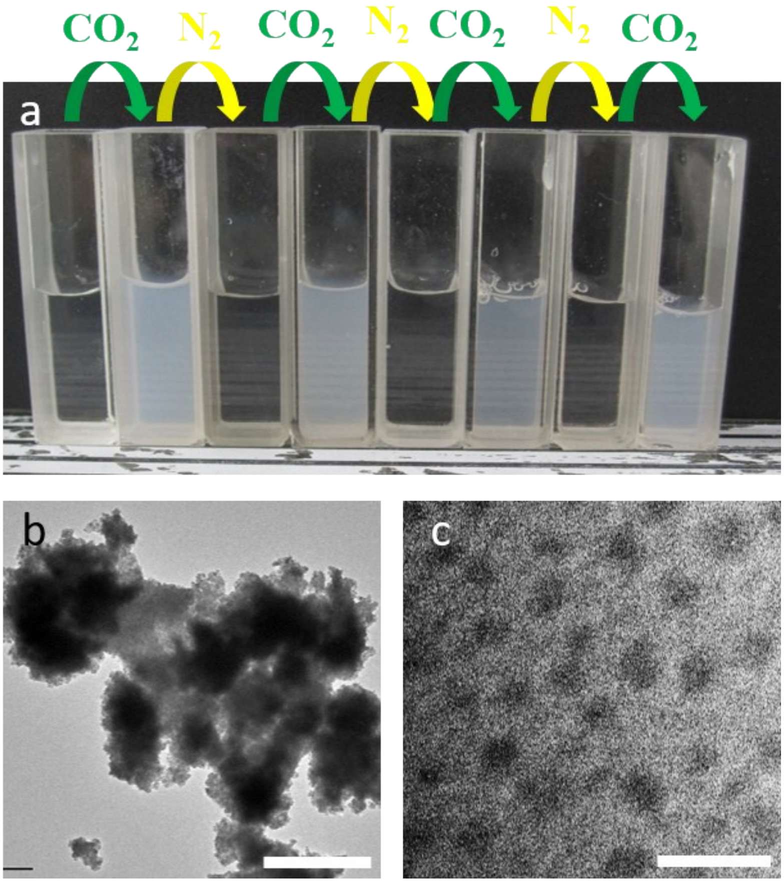

Figure S6. (a) Reversible appearance change of NaCas solution upon cycling between introducing CO_2 and N_2. (b) TEM image of NaCas at pH 4.7, scale bar 500nm. (c) TEM image of NaCas at pH 6.8 after 6 cycles using CO_2/N_2, scale bar 50nm.

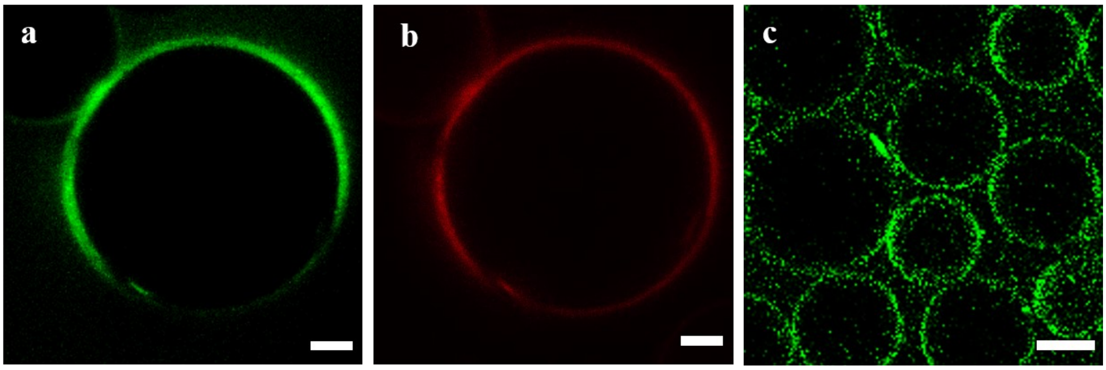

Figure S7. CLSM image of the emulsion stabilized by a mixture of 0.1 wt% NaCas labelled with FITC (a) and 0.5mg mL^{-1} CalB labelled with Cy5 (b). (c) CLSM image of the emulsion stabilized by a mixture of 0.1 wt% NaCas and 0.5mg mL^{-1} lysozyme labelled with FITC. Scale bar 25um (a-b), 100um (c).

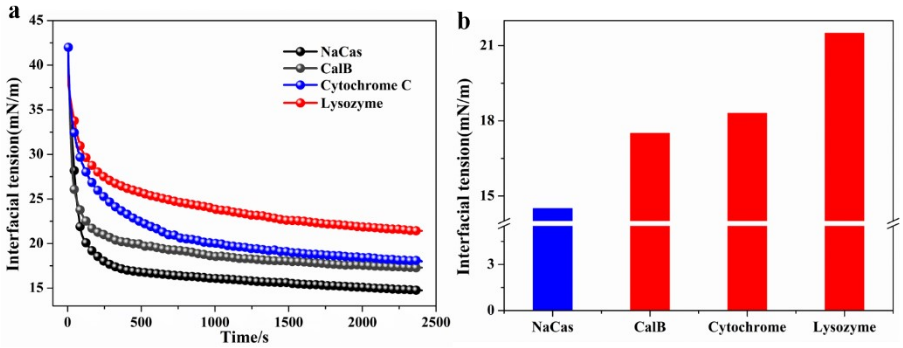

Figure S8. (a) The change in interfacial tension with time using different protein (0.01wt%). (b) Interfacial tension of different protein using dodecane as the oil phase.

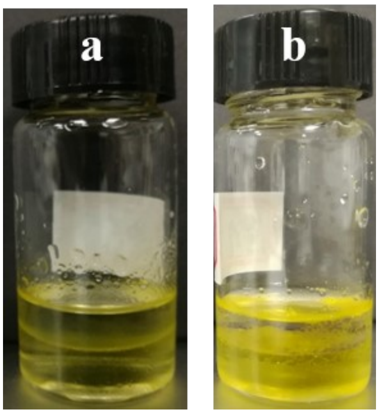

Figure S9. Appearance of emulsions using FITC labeled cytochrome C (0.5mg/mL) as a emulsifer. (b) Appearance of emulsions using FITC labeled lysozyme (0.5mg/mL) as emulsifier. As shown in Figure S6, neither of these two enzymes can form an emulsion.

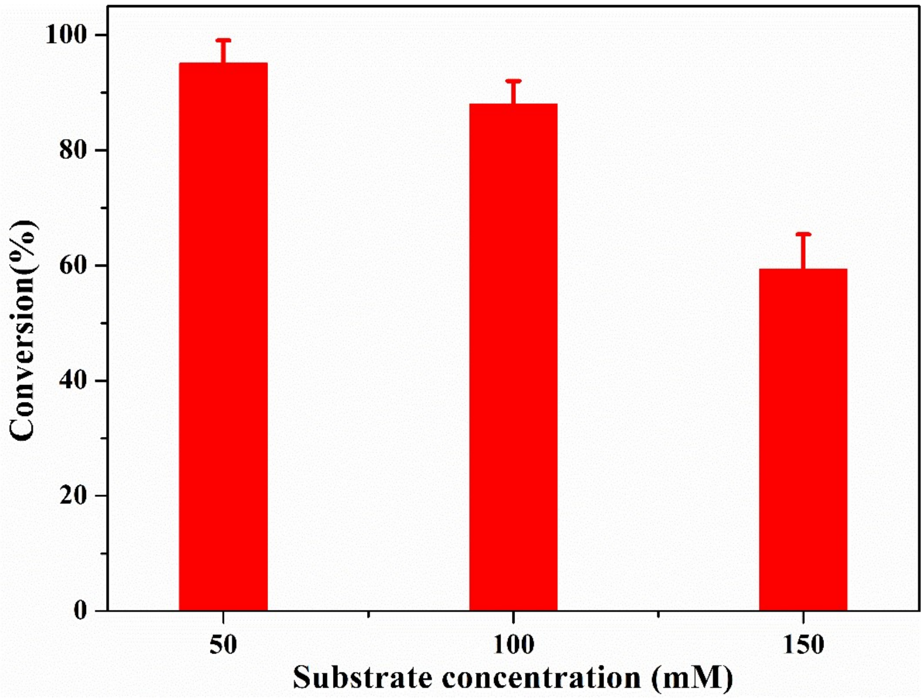

Figure S10. Conversion of the esterification of 1-hexanol with hexanoic acid catalysed by CalB under different substrate concentration.

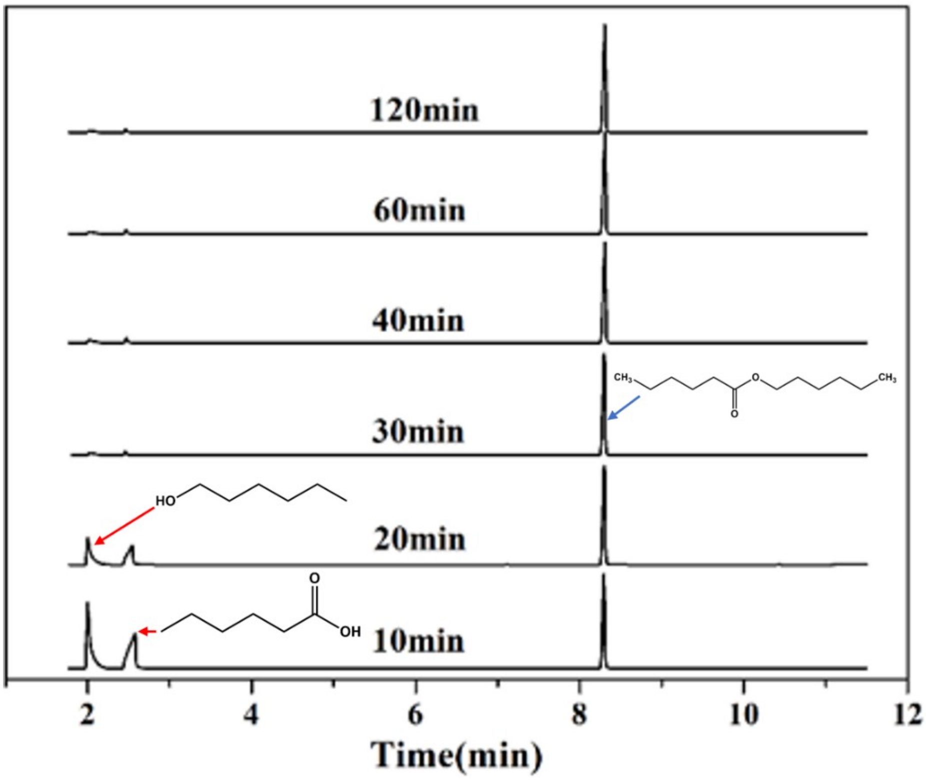

Figure S11. conversion of the esterification of 1-hexanol with hexanoic acid catalysed by CalB under different conditions versus reaction time.

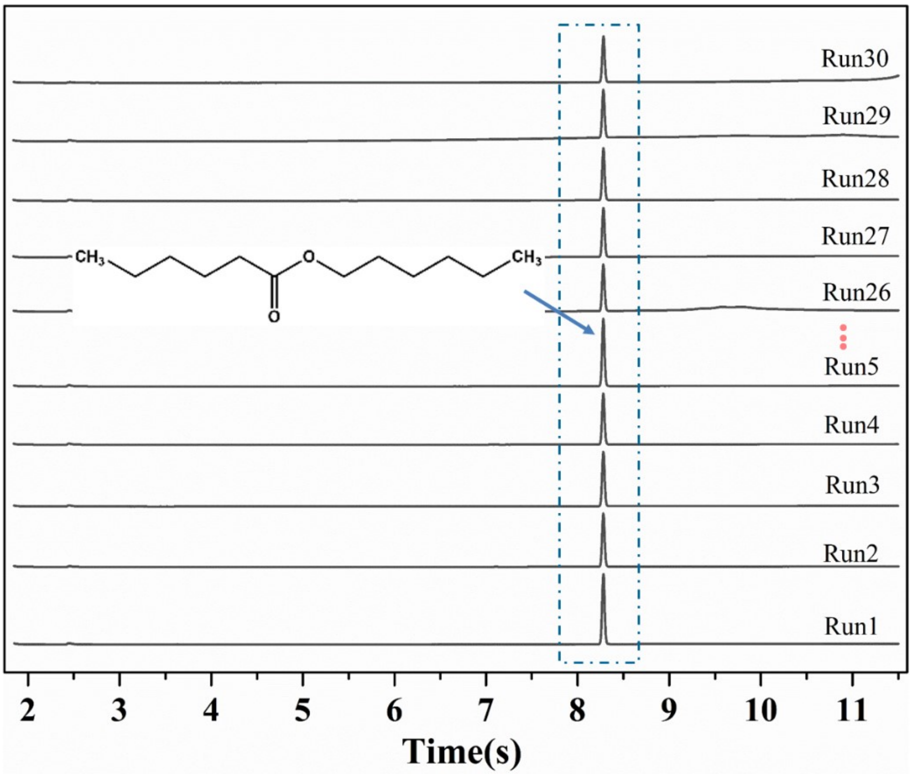

Figure S12. Recycling results for the esterification conversion by interfacial CalB in the N-heptane/water system. The reaction time was 30 min.

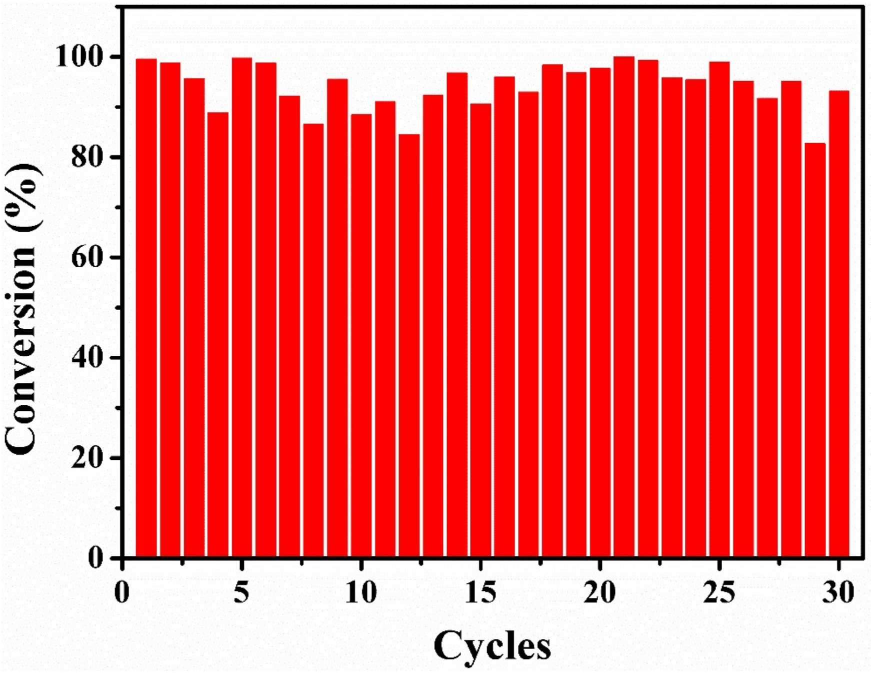

Figure S13. Recycling results for the esterification conversion by interfacial CalB in the n-heptane/water system. The reaction time was 30 min.
